# Supplementary material for: Are carbon emissions trading and green financial instruments synergistic? -Comprehensive quantitative research based on content analysis
Source: PLoS One. 2024 Mar 7;19(3):e0298601. doi: 10.1371/journal.pone.0298601 (PMC10919601; doi:10.1371/journal.pone.0298601)
Supplement: S1 Data — (ZIP) [file pone.0298601.s001.zip › Data/地级市Z_F工作报告.zip!/╕╩╦α/░╫╥°╩╨/└┤╫╘╣1⁄2╓┌║┼í╛┬φ┐╦╩2╛▌═°í┐_2019.docx]

各位代表：

现在，我代表市人民政府向大会作工作报告，请予审议，并请各位政协委员和列席人员提出意见。

2018年工作回顾

过去的一年，在省委、省政府和市委的坚强领导下，我们坚持以习近平新时代中国特色社会主义思想为指导，全面贯彻党的十九大和习近平总书记视察甘肃“八个着力”重要指示精神，以新发展理念为引领，落实高质量发展要求，统筹推进稳增长、促改革、调结构、惠民生、防风险各项工作，较好完成了市九届人大二次会议确定的目标任务。全年完成生产总值511.6亿元，同比增长6.1%；规模以上工业增加值129.03亿元，增长10.2%；社会消费品零售总额219.51亿元，增长7.3%；一般公共预算收入31.07亿元，同口径增长4.9%;城镇居民人均可支配收入29470元，增长7.3%;农村居民人均可支配收入9057元，增长9.6%,经济社会发展迈出新步伐、取得新成效。

——脱贫攻坚成效显著，贫困面貌明显改观。全市上下坚决扛起“百年目标、全党使命”的重大政治责任，始终坚持精准扶贫、精准脱贫，打出“组合拳”，敢啃“硬骨头”，全年减少贫困人口6.31万人，超年度减贫任务1.28万人，贫困发生率由2017年的11.45%下降到6.52%；119个贫困村达到脱贫退出标准，超年度目标任务20个，平川区、景泰县达到脱贫退出标准，申请脱贫摘帽退出，为2020年如期脱贫奠定了坚实基础。

——创新驱动活力迸发，发展后劲切实增强。抓住兰白国家自主创新示范区建设重大机遇，重新布局“一区六园”主导产业，园区定位更加清晰，形成“一区引领、六园联动、多点支撑”的产业集聚发展平台，已成为全市工业经济发展的重要支撑。着力提升科技创新能力，获批建设省级科技成果转移转化示范区，白银科技大市场建成运营，新登记科技成果23项，完成技术市场交易额5.49亿元；新增省级科技企业孵化器2家、省级众创空间3家；新引进两院院士17人，总数达到24人；全市有效发明专利达到353件，排名全省第二位，创新驱动正日益成为白银经济社会发展的强大动力。

——基础设施更加完善，承载能力不断提高。中卫至兰州客运专线全线开工，国道341线白银至中川机场一级公路、景泰至中川机场高速公路、省道217线景泰大水?至白银一级公路、黄河白银段防洪工程等项目加快实施，甘肃中部生态移民扶贫开发供水工程取得突破性进展，白银高新区、银西生物医药产业园、平川陶瓷建材产业园污水处理厂建成运行，刘川新材料产业园升级为省级开发区，综合承载能力进一步增强。

——白银形象大幅提升，城市魅力更加彰显。举全市之力创建全国文明城市和国家卫生城市、园林城市，城市地下综合管廊全线试运行，北京路提升改造基本完成，国土绿化、人居环境整治和拆违治乱强力推进，黄河石林、陶瓷小镇、“六朵金花”、水韵独石、大沟厍弆等旅游景区、特色小镇、美丽乡村更加靓丽、更有内涵，并借助央视《魅力中国城》《美丽中华行》《城市1对1》等栏目高品位宣传白银，提升了城市知名度、美誉度和影响力，城市发展更有色彩、更有亮度、更有温度。

一年来，我们重点抓了以下几项工作：

一、下足绣花功夫抓好脱贫攻坚，全面小康根基更加牢固。把“两不愁、三保障”作为基础性、底线性任务，开展精细精确精微式扶贫，构建了稳定脱贫的四梁八柱。帮扶措施更加精准有效。紧盯贫困人口脱贫6项指标和贫困村退出4项指标，因村因户施策，制定3.82万贫困户“一户一策”精准脱贫计划，开展“点穴式”帮扶。统筹整合涉农资金8.97亿元，争取天津市和平区、西青区东西协作帮扶和中投公司定点帮扶资金1.49亿元，集中用于贫困地区发展产业和改善基础设施。产业扶贫质量明显提升。深入开展“破三弱、强造血”产业扶贫和“两园两率两节点”产业脱贫行动，投入产业扶持资金5.55亿元，贫困村新发展特色农业种植9.96万亩、草食畜养殖13.72万头（只），建成脱贫产业园317个、特色产业园380个，带动贫困户8.69万户，贫困户入园率100%；贫困村新建合作社70家，实现贫困村全覆盖；岷漳移民安置区和靖远、景泰“十三五”第一批光伏扶贫项目并网，带动1.13万户贫困群众增收。基础设施短板加快补齐。完成农村公路“畅返不畅”整治工程388公里，建设农村饮水安全巩固提升工程9处，改造农村危房3515户；2016年和2017年易地扶贫搬迁建档立卡户入住率分别达到100%和97.5%，2018年开工的30个易地搬迁安置点建成住房1663套；新改扩建深度贫困县幼儿园7所，落实5.27亿元资助各类学生36.12万人次；建档立卡贫困人口合规医疗费用实际补偿比达到85%以上，对个人自付合规费用年累计超过3000元的给予兜底救助；贫困人口家庭医生签约服务团队966个，“一人一策”健康措施落实率达到95%以上。

二、加快推进产业结构优化升级，现代产业体系不断完善。坚持有色绿色齐头并进、农业工业协调发展、三次产业融合提升的发展方向，大力培育现代产业体系，加快推进新旧动能转换。传统产业改造提升步伐加快。充分运用国家老工业基地调整改造政策，依托技术创新，延伸产业链条，白银公司铜冶炼技术提升改造和锌冶炼资源综合利用、稀土公司三废深度治理、宏达矿业冶炼废渣综合利用一期等项目建成，昌元化工10万吨重铬酸钠一期、东方钛业钛白粉资源综合利用、恒大陶瓷二期等项目投产，打开了传统产业成长的新空间。战略性新兴产业加速成长。以新能源、新材料、先进装备制造、生物医药、信息技术等产业领域为发展重点，开展优势产业链培育行动，郝氏碳纤维原丝及复合材料、甘肃大象能源年产1万吨电池正极材料、恒丰化工年产2000吨维生素B1生产线、陇草堂年产800吨中药饮片加工等项目建成投产，皓天原料药、阿尔法植物农药原药及中间体等生物医药项目加快推进，战略性新兴产业成为促进产业转型升级的重要动力。现代农业稳步发展。积极谋划农业产业布局，培育壮大特色优势产业和各类经营主体，农产品产供销体系更加完善。落实粮食安全责任制，粮食总产量94.92万吨，增长3.8%。做大特色优势产业，新增瓜菜4.83万亩、果品1.5万亩，蔬菜瓜果等农产品累计外销193.46万吨，实现销售收入46.18亿元；改扩建标准化规模养殖场41个，肉蛋奶产量达到18.82万吨，水产养殖面积达到1万亩；新增道地中药材6.43万亩。做强各类经营主体，引进中天羊业、雄特牧业等大型龙头企业10家，培育省级农业产业化龙头企业17家、省级合作示范社79家，新增家庭农场317家。规模化经营面积达到90.54万亩，占承包地面积的20.9%；农业机械化水平提高1.53个百分点，达到53.53%。打造特色农产品品牌，“三品一标”农产品认证面积达到309万亩，菁茂甘草羊等5个农产品获得国家级农展会金奖，新注册地理标志证明商标15件，靖远县获批筹建全省首个国家地理标志保护产品示范区。现代服务业扩量提质。把文化旅游、商贸物流、金融服务等现代服务业，作为培育新动能的关键领域和推进产业升级的有力支撑，下大力气加以推进。着力打造黄河石林景区，投资3.99亿元的36个配套项目有序推进；成功举办第二届黄河风情文化旅游节、会宁红色旅游文化节等一批重大节会，我市荣获央视“2018《魅力中国城》优秀魅力城市”称号，白银区顾家善村入选《魅力中国城》2018年度魅力乡村旅游目的地和首届“中国农民丰收节”100个特色村庄，景泰县西和村等两个村被评为中国美丽休闲乡村；全年接待游客1337万人次，实现综合收入83.2亿元，分别增长27.8%和29.6%。白银公司综合物流园二期、天奇银东物流园等商贸物流项目加快实施，新增农产品产地批发市场和县乡农贸市场5个，总计达61个，年成交额32亿元。推进电子商务进农村示范县建设，乡镇一级第三方快递网点覆盖率93%，新增电商企业61家、增长28%，网络零售总额7.7亿元、增长30%。优化金融服务，12月末全市金融机构存款余额739.16亿元，增长4.71%，贷款余额655.13亿元，增长3.84%；新增“新三板”挂牌企业1家，新增区域性股权交易市场挂牌企业12家，行政村金融便民服务点覆盖率达到91.2%。

三、紧盯难点堵点推进改革创新，经济发展活力有效激发。充分发挥改革创新的引领作用，以创新倒逼改革，以开放推动创新，着力为经济发展注入新动力、增添新活力。强力推进兰白国家自创区建设，制定自创区“3+2+10”创新驱动政策体系，探索形成“四个依托”创新模式，在规范运行、完善基础、创新突破、强力招商、立体打造等方面持续加力，自创区步入良性发展轨道。白银科技企业孵化器新入孵企业24家，连续三年获评国家级A类科技企业孵化器；新培育高新技术企业17家、省级科技创新型企业25家;科技企业获得兰白技术创新驱动基金子基金股权投资5.78亿元，增信贷款3.84亿元；深化与上海张江等自创区和中科院、兰州大学、兰州交通大学等科研院所交流合作，成功引进甘肃青宇新材料、中检普泰白银医放疗设备检测中心等一批科技成果转化合作项目，全年转化重大科技成果21项。高新区被工信部认定为国家绿色园区，主要经济指标增速均高于全市平均水平，自主创新和辐射带动能力不断提升。进一步深化“放管服”改革，取消调整和下放行政审批事项673项；梳理“最多跑一次”事项1061项，占群众和企业到政府办事事项的82.9%；强化投资项目在线审批监管平台运用，项目限时办结率达到100%；持续推进“多证合一”“证照分离”“企业登记全程电子化”改革，新登记各类市场主体1.7万户，新增注册资本（金）257.69亿元，分别增长17.25%和17.46%。供销合作社综合改革有序推进，乡镇覆盖率达到68%。扎实推进“三去一降一补”，淘汰落后产能52.56万吨，商品房去库存77.07万平方米，刘川新材料产业园列入全国第三批增量配电业务改革试点。坚定不移扩大开放，支持引导白银公司、甘肃品高公司等企业“走出去”，与南非、蒙古、哈萨克斯坦等国家开展合作，甘肃大象能源科技公司被认定为“锂电产业关键材料研发甘肃省国际科技合作基地”。依托白银公司铁运物流、鼎丰现代物流保税监管仓，着力推进中欧国际货运班列常态化运营。引进希望教育、福门休闲生态城等项目110个，签约资金233.4亿元，与省内外34家商会建立稳定合作关系。

四、持之以恒加强生态环境建设，绿色生态底色更加靓丽。践行绿水青山就是金山银山理念，加快推进生态保护和环境治理，城乡生态环境质量稳步提升。着力发展绿色生态产业，制定《推进绿色生态产业发展规划》和十大生态产业专项行动计划，凝练储备的354项绿色生态产业重点项目陆续开工建设，银光集团获得国家绿色系统集成项目资金支持，中材水泥公司入围国家绿色工厂。切实筑牢生态安全屏障，开展“绿盾2018”专项行动，5个省级自然保护区的监管责任全面落实。实施大规模国土绿化行动，重点推进退耕还林、三北防护林等工程和沿黄绿色生态景观带、绿色通道建设，新增营造林33.9万亩。持续推进生态环境治理，开展城乡人居环境整治和拆违治乱行动，清运垃圾88.2万吨，拆除及处理违建25.3万平方米。完成140台燃煤锅炉整治和1.6万户土炕清洁化改造，清理关闭非法煤炭经营户348户，市区空气质量优良天数比例达90.9%；全面落实“河长制”“湖长制”，取缔无证采沙场81家，黄河白银段水质达到Ⅱ类考核目标，县级以上集中式饮用水水源地水质均达到Ⅲ类水质；东大沟河道重金属污染治理等5个土壤污染治理项目加快实施，市区周边生态环境得到有效改善，绿色已成为白银发展的“主色调”。

五、用心用情用力增进民生福祉，人民群众所需所盼进一步满足。全市财政用于民生方面支出136.79亿元，占财政支出比重达83.74%。省、市承诺的民生实事全部办结。就业和社会保障得到加强。新增城镇就业4.41万人，城镇登记失业率2.16%；输转城乡劳动力28.23万人，实现劳务收入54.18亿元。城乡低保标准分别提高7.6%和6.3%，提标后平均保障标准分别达到每人每年5820元和3720元。开工建设棚改安置房6128套，建成1065套。投入270万元实施老年人意外伤害保险补助，提高了老年人及其家庭抵御风险的能力。教育卫生事业全面进步。靖远县通过义务教育均衡发展国家评估认定，“全面改薄”任务基本完成，累计落实资金18.1亿元，建设校舍57.36万平方米，购置2.83亿元设备。建立统一的城乡居民基本医疗制度，医保参保率达到97.8%，大病保险报销比例由50%-65%提高到60%-80%，建立9个县乡村医疗共同体；完成会宁县人民医院、市妇幼保健院综合业务楼等5个市县医疗卫生项目建设。文体事业蓬勃发展。顺利通过国家第三批公共文化服务体系示范区创建验收，开展庆祝改革开放40周年等群众性主题活动500多场次，改造提升贫困村综合性文化服务中心68个，完成永泰城址东墙体、永泰学校修缮、红军会师旧址保护等工程，靖远县平堡村入选第五批全国传统村落名录。策划拍摄的电视剧《淬火成钢》在央视一套黄金时段播出，实现全省历史性突破。乒乓球项目“省队市办”落户我市，代表国家参加塞尔维亚国际青少年乒乓球公开赛取得优异成绩；省第十四届运动会获得金牌34枚，创白银竞技体育最好成绩；成功举办黄河石林踏青自行车赛、国际马拉松赛、全国越野滑雪夏季滑轮锦标赛、全国女子篮球锦标赛、省第九届少数民族运动会等高水平体育赛事。社会大局和谐稳定。安全生产和信访形势总体平稳，食品药品监管不断加强。金融风险稳定可控，社会信用体系建设扎实推进。深化“平安白银”建设，扫黑除恶专项斗争扎实开展，“七五”普法有序推进，公共法律服务体系初步形成，行政村法律顾问实现全覆盖，靖远县庄口村被评为“全国民主法治示范村”。持续推进全国双拥模范城“五连冠”、全省双拥模范城“九连冠”创建工作。我市被命名为全省民族团结进步示范市，白银区被命名为全省民族团结进步示范区，景泰县昌林村被命名为全国民族团结进步示范村。

过去的一年，我们从严从实推进政府自身建设，牢固树立“四个意识”，切实坚定“四个自信”，坚决践行“两个维护”，不折不扣把中央和省委、省政府及市委各项决策部署落到实处。全面推进依法行政，严格执行政府工作规则和重大事项决策程序，主动接受人大法律监督和政协民主监督，90件人大代表建议、172件政协委员提案全部办复。深入开展“转变作风改善发展环境建设年”活动，严格落实省政府“十不准”规定，全市“三公”经费支出下降5.5%。加强审计监督，纠正违规资金1.68亿元。规范公共资源交易，节约财政资金1.42亿元。与此同时，外事侨务、防震减灾、妇女儿童、红十字会、残疾人、住房公积金、地方志、统计、档案、气象、人防等工作也取得了新成绩。

各位代表，过去一年，困难比预想的要大，成绩比预期的要好，全市生产总值首次突破500亿元大关，稳居全省第五位。面对复杂多变的困难挑战，全市上下凝心聚力、滚石上山，在爬坡过坎的艰难跋涉中砥砺前行，过得充实，走得坚定。我们始终坚持践行新发展理念，在负重拼搏中坚定探索具有白银特色的转型发展之路。始终坚持推动高质量发展，在夯基挖潜中着力加快新旧动能转换、构建现代化经济体系。始终坚持深化改革不动摇，在守正创新中不断破解发展瓶颈、培育竞争新优势。始终坚持以人民为中心的发展思想，在聚力攻坚中为全面建成小康社会收官打下坚实基础。始终坚持求真务实工作作风，在团结实干中积蓄起推动白银新时代发展的磅礴力量。

这些成绩的取得，是习近平新时代中国特色社会主义思想定向引航的结果，是省委、省政府和市委正确领导的结果，是市人大、市政协监督支持和社会各界共同努力的结果，更是全市人民凝心聚力、奋力拼搏的结果。在此，我代表市人民政府，向全市各族人民，向各民主党派、工商联、无党派人士、人民团体和社会各界人士，向驻银部队、武警官兵、公安干警和中央、省驻银单位，向所有关心、支持白银改革发展的各界朋友，致以崇高敬意和衷心感谢！

同时，我们也清醒地认识到，全市经济社会发展还面临不少困难和问题。一是脱贫攻坚任务重、时间紧，深度贫困乡村基础设施和公共服务还存在不少短板，产业脱贫的长效机制还没完全形成。二是经济企稳回升的基础尚不牢固，固定资产投资增长乏力，好项目、大项目储备较少；一些企业生产经营困难，减停产企业较多；偿债压力巨大，新增财源不足，财政收支矛盾突出。三是科技创新投入不够，企业创新主体作用发挥不充分，高科技领军人才、现代企业经营管理人才、拥有新理念的复合型管理人才和高技能人才匮乏。四是生态环境相对脆弱，历史遗留的生态环境问题突出，大气、水、土壤污染防治和国土绿化、全域无垃圾治理及绿色生态产业体系构建任务艰巨。五是基础设施仍然薄弱，尚未形成立体高效的交通体系，大型水利工程设备严重老化，园区配套及服务体系还不完善。六是营商环境还需进一步优化，个别部门和干部服务意识不强、担当不够，中小微企业“融资难”“融资贵”问题突出。我们将直面这些发展中的困难和问题，努力在今后的工作中采取针对性的措施，认真加以解决。

2019年奋斗目标和重点工作

今年是新中国成立70周年，是全面建成小康社会关键之年。做好今年的工作，意义重大。研判当前经济社会发展形势，既要清醒看到经济运行稳中有变、变中有忧的严峻一面，也要看到国家将进一步稳就业、稳金融、稳外贸、稳外资、稳投资、稳预期，围绕高质量发展、提振消费、新型基础设施建设、乡村振兴、民营企业发展等方面出台一系列利好政策；省上加快构建绿色生态产业体系，全力支持兰白国家自创区建设，出台产业发展基金、科技创新先行先试等一揽子政策措施，这些都必将为我市高质量发展提供难得机遇。面对复杂多变的经济形势，只要我们牢固树立辩证思维和底线思维，增强忧患意识，坚定发展信心，全面贯彻落实中央和省委、省政府推动高质量发展的决策部署，用足用好区位优势、黄河优势和大工大农基础优势“三个优势”，着力打造兰白都市圈率先发展、全省三次产业融合发展示范、西部科技创新发展示范“三个高地”，尽力突破贫困、基础、生态、科技、人才等制约发展的“五个瓶颈”，努力做强优势工业、高效农业、特色旅游、工业物流和宜居宜业宜游宜养山水城市建设“五个领域”，就一定能够保持经济持续健康发展和社会大局稳定，为全面建成小康社会收官打下决定性基础。

今年政府工作的总体要求是：坚持以习近平新时代中国特色社会主义思想为指导，全面贯彻党的十九大和十九届二中、三中全会精神，深入落实习近平总书记视察甘肃重要讲话和“八个着力”重要指示精神，统筹推进“五位一体”总体布局和协调推进“四个全面”战略布局，按照中央经济工作会议和省委十三届七次全会、市委八届八次全会部署要求，坚持稳中求进工作总基调，坚持新发展理念，坚持推动高质量发展，坚持以供给侧结构性改革为主线，坚持深化市场化改革、扩大高水平开放，突出抓好十大绿色生态产业，继续打好三大攻坚战，统筹推进稳增长、促改革、调结构、惠民生、防风险工作，增强人民群众获得感、幸福感、安全感，加快建设繁荣富裕、文明和谐、美丽宜居的现代化新白银。

经济发展主要预期目标是：生产总值增长6％，规模以上工业增加值增长4.5%，固定资产投资增长6％，社会消费品零售总额增长8％，一般公共预算收入增长6％，城镇居民人均可支配收入增长7％，农村居民人均可支配收入增长8％，居民消费价格指数涨幅控制在3％以内。

为实现上述目标，重点做好六个方面的工作：

一、紧盯短板弱项，坚决打赢打好精准脱贫攻坚战。坚持把脱贫攻坚作为全面建成小康社会的底线任务和高质量发展的首要任务，精准施策，强力攻坚，确保会宁、靖远两个深度贫困县脱贫摘帽，144个贫困村脱贫退出，减少贫困人口6.68万人，贫困发生率下降到1.29%。

着力提升产业扶贫质量。按照“独一份、特别特、好中优、错峰头”的思路，用足用好特色产业发展工程贷款、农产品收购贷款和到户产业扶持资金，大力发展牛、羊、蔬、果、薯、药、小杂粮、黑毛驴、水产养殖等特色产业，确保村有主导产业、户有增收渠道。加快完善生产组织、投入保障、产销对接、风险防范四大体系，总结推广“中天模式”“海升模式”，探索华西希望集团产业扶贫模式，引进和培育骨干龙头企业50家。实施农民专业合作社能力提升工程，创建省级示范合作社80家左右，其中贫困村60家左右;推广“宕昌模式”，鼓励有实力的合作社控股组建富民公司，形成“产供销”一条龙的产业链、利益链。引导企业兴办“扶贫车间”70个。每个行政村建立金融综合服务室，全面开办种养产业综合保险，支持县区开办“一县一（多）品”特色保险品种，提高保险覆盖率。巩固和拓展农产品出省工程，加快优质特色农产品走出去。实施贫困乡“互联网+”产业扶贫行动，培育贫困地区农产品网货品牌和本土电商平台，搭建农产品“线上线下”交易平台，扩大特色农产品网销规模。实施光伏扶贫工程，建成59.1兆瓦光伏扶贫电站，带动9303户建档立卡贫困户增收。

着力改善基础保障条件。加快实施农村饮水安全巩固提升工程和水窖水净化处理，农村集中供水率和自来水普及率分别达到85%和82%，确保群众喝上稳定安全饮用水。全力推进易地扶贫搬迁，完成2018年2998户1.27万人的搬迁任务。加强“四好农村路”建设，完成“畅返不畅”整治工程195公里。加快推进新一轮农村电网改造升级和动力电向村组延伸。深入推进教育扶贫，实施农村小规模学校、寄宿制学校和教师周转宿舍等项目建设，着力改善农村学校办学条件。全面推进健康扶贫工程，继续开展“三个一批”行动，巩固扩大“一保四有”成效，大病救治病种扩大至23种，建档立卡贫困户个人自付合规医疗费用年累计控制在10%-15%。

着力激发脱贫攻坚内生动力。坚持扶贫与扶志、扶智相结合，引导贫困群众树立主体意识，克服等靠要思想，切实增强勤劳致富的主动性和自信心。开展订单式、定向式实用技术和劳动技能培训，培训建档立卡贫困劳动力0.91万人，开发乡村公益性岗位1235个。实施“精神扶贫”工程，选树一批脱贫致富先进典型，总结推广成功经验，用身边人身边事教育引导群众，推动形成自力更生、脱贫光荣的鲜明导向。

二、加快产业升级，推进绿色发展崛起迈出新步伐。着力把生态产业作为培育新动能的重中之重和高质量发展的主攻方向，加快产业结构优化升级，打造全省三次产业融合发展示范高地。

进一步改造提升传统产业。立足大工业基础，以新技术、新业态推动有色金属、化工、能源等传统产业改造升级，促进节能环保、清洁生产、清洁能源产业发展。节能环保产业方面，持续推进循环经济示范城市建设，加快凹凸棒石资源开发，开工新奥综合能源站、西部恒生年处理60万吨废铅酸蓄电池、银光公司危险废物处置等项目，建成白银公司湿法炼锌渣综合回收、昌衍汽车回收拆解及废旧物资回收利用等项目。清洁生产产业方面，实施白银公司绿色化改造工程，开工永能年产6万吨再生铅、诺尔达硅酸钙板生产线等项目，建成远昊年产6万吨岩棉制品生产线等项目。清洁能源产业方面，抓住国家配额制等政策契机，落实直购电交易、降低企业用电价格等系列措施，提高新能源就地消纳比例，提升外送电量，有效降低全市弃风弃光率。

加快培育战略性新兴产业。先进制造产业方面，加快宏达铝型材年产5万吨铝型材、容和集团矿用防爆电器智能制造生产线、新乐雅年产500万平方米喷墨渗花抛光砖、北鋀生物科技全球新药生产服务基地、康奇年产1万吨医药中间体等项目建设进度，力争白银大象年产5000吨锂电池正极材料生产线项目建成投产。中医中药产业方面，持续扩大道地药材标准化种植面积，提升道地药材质量，打造白银中医药产业品牌；引进和培育一批加工型、营销型龙头企业，加快仁博药业中药材产业园、会宁杨集中药材产业园等项目建设，推动形成中药材种植、加工、研发、物流、仓储、贸易等全产业链。数据信息产业方面，推进新型智慧城市建设，加快“城市通”平台的开发应用，实现数据资源交换共享、互联互通，消除“信息孤岛”；加快白银市工业云平台、“智慧园区”、鑫海成大数据中心等项目建设，促进工业物联网和工业大数据集成应用；主动参与省上国际互联网数据专用通道项目建设，提升白银公司等重点骨干企业国际互联网数据传输性能。军民融合产业方面，加快银光公司5000吨特种PC生产装置项目建设，建成郝氏碳纤维循环应用产业示范园一期，推进光气化、氟化工、民爆、碳纤维等4个军民融合产业“园中园”建设，打造军民融合创新示范区。通道物流产业方面，立足区位优势，加快推进白银公司综合物流园二期、银东工业物流园、刘川工业物流园、景泰物流仓储汽配园、邦农现代农业物流园等重点物流园区建设，不断提升货运吞吐量、仓储能力和装卸能力，努力把白银打造成甘肃乃至西部的工业物流集散中心。以白银公司公铁联运和鼎丰综合保税物流园为依托，积极融入中新南向通道，推动中欧国际货运班列常态化运行，支持白银公司等国有骨干企业和有实力的非公企业“走出去”，带动特色农产品、中医药、陶瓷等特色产品进入国际市场。

大力发展绿色富民产业。文化旅游产业方面，围绕建设省内一流国内知名优秀旅游城市目标，积极推进旅游强市建设，着力打造“一个龙头景区，四条精品线路”，加快构建“龙头带动、多点开花、网状辐射、全域推动”的旅游发展新格局。坚持景区“大旅游”和乡村“小旅游”一起抓，重点做好黄河石林5A级景区创建工作，推进黄河石林国际露营地、水川湿地公园二期、金沟河生态景观带、水川康养小镇等项目建设，开发生态观光、乡村休闲、农耕体验、创意农业等为主的新业态，建设一批乡村旅游景区、示范村。挖掘黄河文化、工矿文化、红色文化、丝路文化、民俗文化等资源，创作一批文旅创意舞台剧等精品节目，开发铜艺制品、陶艺制品、刺绣、剪纸等特色旅游商品，提升旅游品质和附加值。开通白银旅游专线，畅通旅游景区景点、重点乡村旅游节点骨干网络，形成大旅游交通环线。注重全季节体验，推出春赏花、夏消暑、秋采果、冬玩雪旅游项目，丰富旅游产品供给，满足游客多样化旅游需求，努力把旅游业打造成绿色发展的支柱产业和综合性幸福产业。循环农业方面，充分发挥农业基础优势，加快完善“一带两区六基地”现代农业布局，大力推广绿色高效现代都市农业循环模式、粮畜果一体化区域循环模式和戈壁生态农业循环发展等新模式，着力提升农业发展质量和生态服务功能，新增“粮改饲”面积5万亩，农产品加工转化率达到59%，废旧农膜回收利用率达到80.2%，畜禽养殖废弃物综合利用率达到73%，秸秆综合利用率达到83%，实现农药化肥使用零增长。推进白银国家农业科技园区和靖远、景泰国家级现代农业产业园建设，加快德康300万头生猪养殖及加工、阿西娅生态牧场及西北滩羊育种基地、会宁中天羊业肉羊精深加工、海大集团景泰万亩南美白对虾养殖等项目进度，举办第五届（2019）中国驴业发展大会暨第三届国际毛驴产业发展论坛，推动现代农业规模化、特色化、品牌化。加快发展农产品冷链物流，完成果蔬保鲜库28座，购置移动冷藏车14台，新增贮藏能力1.5万吨以上，着力形成覆盖生产基地到消费终端的全链条服务体系。

三、强化动能支撑，着力厚植高质量发展新优势。始终把创新摆在高质量发展的核心位置，以项目建设为支撑，最大限度发挥投资的关键作用，推动有质量、有效益、可持续的发展。

大力实施创新驱动战略。立足打造西部科技创新发展示范高地目标，坚持“集聚产业、输出科技、研究实践、循环互用、精准错位”的功能定位，加快推进兰白国家自创区建设，落实自创区“3+2+10”创新驱动政策，激发各类创新主体活力，加快打造科技体制改革试验区、产业品质跃升支撑区、人才资源聚集区、东西合作发展先行区和生态文明建设引领区，推动“一区六园”向更高层次更高水平发展。加强科技创新平台建设，完善“众创空间+孵化器+加速器+产业园”梯次孵化体系，新增一批众创空间、星创天地等创新平台，创建技术创新中心3个，引进建设产业研究院1-2家，实施科技成果转化项目20项，新增知识产权试点县区1-2个、知识产权优势单位2-3家。强化企业创新主体地位，加大财税、信贷、用地等支持力度，培育高新技术企业10家以上、科技型企业15家以上。实行更加积极、开放、有效的人才政策，用好用活院士、归根人才、本土人才“三支队伍”，强化人才服务保障，构建新时代白银人才“联合舰队”。

切实加大招商引资力度。以承接中东部产业转移、积极参与“一带一路”建设为契机，强化招商引资工作，着力提高招商实效，为高质量发展注入更多源头活水。瞄准华为、腾讯等世界500强、全国500强、民营500强企业，有针对性地开展产业链招商、点对点招商、园区招商，引进一批科技含量高、带动能力强、税收贡献大的好项目、大项目。全年计划招引项目100个以上，签约资金达到230亿元以上。全面落实招商项目跟踪责任制，以项目落地论英雄，对签约的项目，持续攻关，搞好服务，打通审批“绿色通道”，提高招商引资合同履约率、项目开工率和资金到位率，确保早落地、早投产、早见效。

着力推进重大项目建设。开展重点项目建设“提速增效”推进行动，实施市列重点项目143个，年度完成投资170亿元。开工建设景泰永泰川农业灌溉引水工程、白银至青城古镇旅游公路、国网白银通用机场等项目，特别要全力保障全市人民期盼已久的甘肃中部生态移民扶贫开发供水工程早日开工；加快中兰客专白银段、国道341线白银至中川机场一级公路、景泰至中川机场高速公路、省道217线景泰大水?至白银一级公路、省道209线郭城至巉口段及共和至黑城子段公路、靖远金滩黄河大桥和靖乐渠环境综合治理等项目进度。推进环海中铁路、白银至兰州站高铁、国道341线辘轳坝至白银段、省道35线景泰至礼县高速公路白银段、省道103线沿黄快速通道、省道315线景泰至山丹公路景泰段、省道225线会宁至华家岭公路等重大项目前期工作。加大亚行贷款黄河上游白银段生态修复及可持续发展、世行贷款黄河石林大景区文化自然遗产保护与开发等外资项目争取力度，借助外力推动全市经济社会发展。

积极支持民营经济发展壮大。认真落实国家和省上促进民间投资的政策措施，鼓励和引导民间资本进入交通、教育、医疗、养老等领域。严格落实国家支持民营企业发展壮大6个方面的政策举措和省上支持中小微企业高质量发展的58条政策措施，毫不动摇地鼓励、支持、引导民营经济发展。强化融资担保体系建设，将小微企业互助担保政府风险补偿金基金池由3000万元扩充到5000万元，提高土地、房产、林权、知识产权等抵押率，着力破解民营企业融资难题。积极构建“亲”“清”新型政商关系，畅通政银企沟通渠道，着力营造依法保护市场主体合法权益的法治环境、公平竞争的市场环境、鼓励创业的社会环境。弘扬优秀企业家精神，大力支持创新创业，表彰奖励一批优秀企业家，真正让创业者受尊重、企业家有地位。

四、坚持生态优先，加快建设宜居宜业宜游宜养新家园。始终把绿色作为高质量发展最靓丽的底色，持续推进“三城同创”，改善生态环境，让白银的天更蓝、水更清、地更净、风景更美。

着力提升城市品位。发挥规划引领作用，修编白银城区银西片区、旧城区控制性详细规划，编制金沟两侧、高铁站周边用地控制性详细规划，推进全市“多规合一”编制工作。加快城市道路、供热、供水、排水、园林等基础设施建设，新续建诚信大道北段、长春路西段、银山路等道路10条，改造提升万盛路等道路3条、公园2个，新建人行天桥1座，不断增强城市承载能力。加快污水处理设施建设，市区污水处理率达到95%以上，会宁、靖远、景泰三县污水处理率达到85%以上，力争11个重点建制镇污水收集处理设施开工建设，利用净化处理后的工业废水打造多湖景观，做足做活城市“水”文章。继续推进保障性安居工程建设，改造老旧小区“三供一业”住宅楼1500栋、面积480万平方米。以城区东出口、国道109线城区过境段为重点，加大环境综合整治力度，着力改善城区出入口面貌。完善城市管理体系，推广“街长制”管理模式，靠实网管员监管责任，提升城市精细化管理水平。

加快实施乡村振兴战略。坚持农业农村优先发展，完善乡村振兴战略规划，因地因村编制乡村建设整治规划，优先推进沿黄美丽乡村示范带建设，建成省市级美丽乡村示范村15个。推进田园综合体和特色小镇建设，确保每个县区至少有1个田园综合体和1个特色小镇。持续开展农村人居环境专项整治行动，完成农户卫生改厕6.81万户，新改建城乡公厕600座。开展“美丽庭院”创建，推广“四净两规范”做法，美化净化生活环境。实施村庄造林绿化行动，新增村庄绿化面积2.5万亩。推进新型职业农民培育工程，培育“土专家”“田秀才”，提升农村人才科技素质。扎实推进农村信用体系建设，着力改善农村金融生态环境。建立健全村民共建共治体系，开展农村社区建设示范创建活动，推行“三议五会”工作法，提升农村治理水平。大力开展移风易俗和丰富多彩的乡村文化活动，遏制天价彩礼、大操大办、厚葬薄养等陈规陋习，着力培育文明乡风、良好家风、淳朴民风。

打好污染防治攻坚战。认真落实中央和省环保督察反馈意见，加快黄家洼区域生态恢复治理等问题的整改。坚决打好蓝天、碧水和净土保卫战，开展工业企业污染治理，实施火电等重点行业超低排放改造，推进燃煤锅炉综合整治，确保市区空气质量优良天数比例达到83%以上。全面落实河长制，推进祖厉河等重点流域水污染整治，完成县级以上集中式饮用水水源地规范化整治。加快东大沟河道重金属污染治理、白银历史遗留含铬土壤污染治理等工程进度，开工建设银光公司土壤硝基化合物污染治理工程。继续推进大规模国土绿化行动，完成营造林32万亩以上。开展“绿盾2019”专项行动和环境问题大排查大整治，保持打击环境违法行为高压态势。

五、持续深化改革，有效激发经济社会发展新活力。坚持把改革作为推动高质量发展的关键一招，以新发展理念为引领，切实破除阻碍发展的瓶颈制约。

着力推进供给侧结构性改革。巩固和扩大“三去一降一补”成果，推动更多过剩产能加快出清，依法依规处置“僵尸企业”，为绿色生态产业腾出资源、市场和发展空间，推进形成生产要素从低质低效领域向优质高效领域流动机制，发展更多优质企业。认真落实好减税降费政策措施，进一步清理和规范涉企收费，积极做好刘川新材料产业园国家增量配电业务改革试点工作，争取在降低实体经济成本方面有更大突破，切实减轻企业负担，不断增强企业竞争力。

持续推进“放管服”改革。树立审批服务就是生产力、就是软实力、就是具体项目的理念，最大限度简化审批程序，最大力度提供优质服务。推进“一窗办、一网办、简化办、马上办”改革向乡镇街道、村社区延伸，实现“最多跑一次”市县乡全覆盖。加快“互联网+政务服务”建设，压减办事流程和环节，市县两级政务服务事项网上可办率达到90%以上；完善提升“一窗受理、集成服务”，推动综合性政务大厅转型升级，实现政务服务事项应进必进、“一扇门”办理。持续开展“减证便民”行动，畅通网上咨询投诉渠道，切实解决群众在政务服务中遇到的困难和问题。全面推行统一的市场准入负面清单制度，全域实行企业投资项目承诺制、代办服务、模拟审批、容缺受理和建设项目区域化评估评审等优化项目审批服务的措施办法。推进“企业开办全程网上办”改革试点工作，允许“一照多址”“一址多照”，打破市场主体住所和经营场所限制。强化“双随机、一公开”监管，实现“一次抽查、全面体检”，提高监管成效。

深入推进重点领域改革。深化投融资体制改革，积极争取政府债券，拓宽基础设施项目资金来源渠道，降低政府融资成本。持续推进农村集体产权制度改革及“三变”改革，完成农村集体资产清产核资工作，健全产权交易和金融服务平台，搭建产权评估和担保平台，不断完善农村产权流转交易市场，开设乡镇农村产权交易窗口和村级服务点。进一步深化集体林权制度配套改革，稳步推进天然气价格改革和农业水价综合改革。全力推进全市党政机构改革工作，确保按期全面完成改革任务。加强政府债务限额管理，遏制债务增量，稳妥化解债务存量，进一步巩固金融秩序，确保不发生区域性金融风险。深化供销合作社综合改革，实施乡村加油站建设项目，方便农民群众用油。

六、突出民生保障，不断提高人民群众获得感幸福感安全感。坚持以保障和改善民生为根本，始终把人民利益摆在至高无上地位，加大民生投入，增进民生福祉，让改革发展成果更多更公平惠及人民群众。

着力提升社会保障水平。扎实推进全民创业，城镇新增就业4.1万人以上，城镇登记失业率控制在4%以内。实施全民参保登记计划，做好中小企业从业人群、灵活就业人员等重点人群参保工作，全民参保登记率达到90%以上。继续深化养老保险制度改革，扩大失业保险稳岗补贴发放范围，稳妥推进跨省异地就医直接结算，做好社会保险待遇提标工作。开展欠薪隐患排查化解，治理拖欠农民工工资问题。做好退役军人工作，维护军人军属合法权益。全面实施居家养老服务，持续提升养老服务质量。实施残疾人精准康复服务行动，确保有康复需求的残疾儿童和持证残疾人康复服务率达到80%以上。落实各项社会救助政策，提高城乡低保标准和补助水平。抓好农村留守儿童关爱保护和困境儿童分类保障工作。

切实办好公平优质教育。全面贯彻落实全国教育大会精神，持续深化教育综合改革，解决阻碍教育发展的顽瘴痼疾。加快推进希望集团甘肃职业技术学院、市特殊教育学校教学楼和宿舍楼、会宁县红军小学、亚行贷款靖远职专、景泰一中整体搬迁、平川雄立职业技术学校等项目，努力改善各级各类学校办学条件。加快学前教育普惠发展，加大普惠性民办幼儿园扶持力度，支持农村地区和贫困地区新建、改扩建幼儿园。推进义务教育优质均衡发展，促进义务教育学校标准化管理，做好控辍保学工作，着力解决“择校热”“大班额”等突出问题。办好特殊教育、继续教育和民办教育，优质发展高中阶段教育，提升职业教育办学水平。全面落实乡村教师生活补助，妥善解决代课人员问题，扎实做好教师待遇落实工作。

加快推进健康白银建设。健全完善现代医院管理制度，着力推进公立医院薪酬制度改革试点、紧密型医联体建设试点工作和基本医保支付方式改革，巩固完善分级诊疗制度，组建白银市医疗集团，完善县乡村一体化管理，促进优质医疗资源整合共享、下沉基层。加快全民健康信息平台建设，开展“互联网+医疗健康”服务，实现电子健康卡“一卡通用”。实施公共卫生服务能力提升行动计划和“优质服务基层行”活动，推动基层服务能力提质达标。落实重大疾病防控措施，进一步提升精神卫生、职业病防治服务能力。推进中医药产业和医养结合发展，实施中医药人才培养计划，建设一批优势突出、特色明显的中医队伍。

繁荣发展文体事业。深化国家公共文化服务体系示范区创建工作，广泛开展庆祝新中国成立70周年系列文艺活动，实施文化惠民工程，推出一批有本土特点、体现时代精神、传递正能量的精品力作，不断丰富城乡群众文化生活。启动应急广播建设工程，加强贫困地区县级广播影视机构制播能力建设。抓好市县级融媒体中心建设，构建集宣传报道、信息发布、便民服务为一体的融合传播平台。推进文物遗产保护传承，加快会师旧址防雷工程和景泰明长城、永泰城址保护等项目建设。实施农村体育基础设施提升工程，推动政府公共体育设施及各企事业单位、学校所属体育场馆向社会免费或半免费开放，逐步实现资源共享。加快推进白银国家冬季运动项目训练基地建设，开展体育赛事经营活动，引进和承办国家级、省级高水平体育赛事，着力打造一批品牌赛事。

不断推进社会治理创新。深入开展“七五”普法，规范公共法律服务平台运行，落实法律顾问制度，强化“谁执法谁普法”责任制落实。抓好重点行业领域安全监管和专项整治，进一步提升应急救援能力，坚决防范遏制重特大事故发生。加强食品药品安全监管，保障人民群众饮食用药安全。推进“雪亮工程”和“智慧警务”建设，持续开展扫黑除恶专项斗争，严厉打击各类违法犯罪活动，有效维护社会和谐稳定。深化信访制度改革，坚持好发展好“枫桥经验”，有效化解矛盾纠纷和信访积案。贯彻落实党的民族政策和宗教基本方针，争创全国民族团结进步示范市，巩固和发展民族团结、宗教和顺的良好局面。

认真办好几件民生实事。（1）为全市69个乡镇卫生院各购置1台远程心电检查设备。（2）对20所采用燃煤锅炉和小火炉供暖的农村中小学校采暖设施进行改造。（3）实施棚户区改造5267套。（4）实施农村危房改造1541户。（5）为全市57万户常住居民办理自然灾害救助保险。（6）实施百名创客引领计划。（7）实施“百场乡村春晚”演出计划。（8）实施行政村（社区）体育健身工程66个。

新时代必须有新担当新作为，也对政府自身建设提出新的更高要求。我们一定要把抓落实作为政府工作的生命线，干字当头，敢于担当，创造性地推动各项目标任务落实。

坚持忠诚为政。旗帜鲜明讲政治，把党的领导贯穿政府工作全方位、全过程，树牢“四个意识”，坚定“四个自信”，践行“两个维护”，自觉用习近平新时代中国特色社会主义思想武装头脑、指导实践、推动工作，确保中央和省委、省政府及市委各项决策部署落地见效。

坚持依法行政。全面加强法治政府建设，带头尊法、学法、守法、用法，依照法定权限和法定程序行使权力、履行职责，把政府各项工作全面纳入法治轨道。自觉接受人大法律监督、政协民主监督，认真办理好建议提案。大力推进政务公开，让行政权力在阳光下运行。

坚持实干勤政。以钉钉子精神抓好工作落实，特别是在脱贫攻坚、污染防治、自创区建设、招商引资等重点工作的主战场，勇往直前，一抓到底，定一件干一件，干一件成一件。树立干事创业的良好导向，健全正向激励机制和容错纠错机制，给干事者鼓劲、为担当者撑腰，营造想干事、敢干事、能干事、干成事的良好氛围。

坚持廉洁从政。认真落实全面从严治党要求，自觉履行“一岗双责”，始终把纪律和规矩挺在前面，严格执行中央八项规定实施细则和省市委相关规定，坚决反对形式主义、官僚主义，减轻基层负担。强化审计监督，纵深推进政府系统党风廉政建设和反腐败工作，让清正清廉清明成为政府的鲜明底色。

各位代表！时代赋予重任，奋斗创造幸福。新一年的蓝图已经绘就，冲锋的号角已经吹响。让我们更加紧密地团结在以习近平同志为核心的党中央周围，全面贯彻落实中央和省委、省政府各项决策部署，在市委的坚强领导下，坚定信心，锐意进取，攻坚克难，埋头苦干，为决胜全面建成小康社会而努力奋斗，以优异成绩向新中国成立70周年献礼！
